# Bugs as Drugs, potential self-regenerated innovative cancer therapeutics approach for global health

**DOI:** 10.7189/jogh.10.010311

**Published:** 2020-06

**Authors:** Luis Alejandro Salicrup, Miguel Ossandon, Ben Prickril, Avraham Rasooly

**Affiliations:** 1 National Cancer Institute, Center for Global Health, Rockville, Maryland, USA; 2 National Cancer Institute, Division of Cancer Treatment and Diagnosis, Rockville, Maryland, USA

According to estimates from the International Agency for Research on Cancer (IARC), in 2018 there were 17.0 million new cancer cases and 9.5 million cancer deaths worldwide [1]. Cancer is rapidly becoming a major health care problem, especially in low- and middle-countries (LMICs), where 60% of the world’s total new cases are diagnosed [2]. **Figure 1** shows a higher number of cancer deaths rate in countries with low human development index (HDI) compared to countries with very high HDI. HDI is a relevant parameter developed by the United Nations Development Program (UNDP) that provides an overall index of economic development. LMICs face challenges and opportunities in spending scarce resources on cancer prevention and treatment. Cost, access to care, manpower and training deficits, and a lack of awareness in the general population and medical communities are examples of some of the challenges faced by LMICs [3]. Microbial-based cancer therapy may offer an opportunity to address the issue of global cancer therapy disparity and introduce more suitable cancer immunotherapy approach to LMICs.

**Figure 1:**
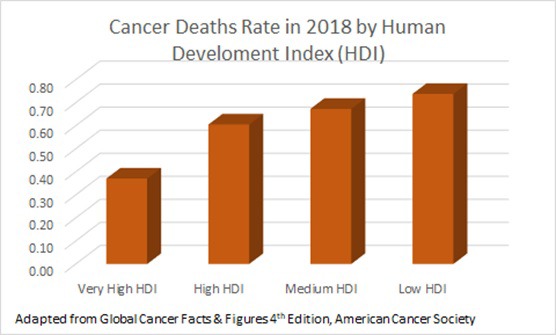
Cancer deaths rate according to Human Development Index.

Cancer therapeutics are not widely available, nor accessible to most patients in LMICs because cancer surgery, chemotherapy, and radiotherapy required medical expertise, which are not widely available to deliver and follow up treatment. The majority of cancer therapeutic equipment is sophisticated, costly and require technical infrastructure to maintain and operate. Most cancer drugs are expensive and unaffordable. While conventional cancer therapy may be available in LMICs urban centers for some of the population, it may not be available in rural areas where a significant part of the population lives. So, current cancer therapy approaches cannot address the clinical needs and are not sustainable for the majority of the population in LMICs and there is an urgent need to develop innovative and sustainable cancer therapeutic approaches to serve the growing needs for cancer therapeutics for global health.

## TRENDS IN CANCER THERAPY

In the last few years, cancer research has expanded to include a more thorough exploration of immunotherapy modalities with the introduction of monoclonal antibodies, immune checkpoint blockers. In the last two years, CAR-T cell therapies individualized therapy which engineer the patient own immune system to treat hematologic cancers were introduced. While such effective cancer therapeutics demonstrate the potential of immunotherapy, these therapies are exceptionally expensive (eg, ~ US$400 000 per single patient CAR-T treatment) while the annual health care expenditure in low-income countries such Burkina Faso, was US$82 per capita a year in 2016. Paradoxically, such prohibitively expensive immunotherapies may prompt to develop affordable cancer immunotherapies across the world and especially for LMICs.

Indeed, the first non-surgical cancer known as the Coley’s toxins therapy, introduced in 1891 [4], was bacterial immunotherapy to treat solid tumors. However, because in many cases it was harmful and ineffective, and with the emergence of radiotherapy and chemotherapy, it was discontinued. However, more recent research suggest that microbial cancer therapy has the potential to address conditions where conventional cancer therapies are inadequate. Anaerobic bacteria including *Salmonella*, *Escherichia coli, Clostridium* or *Bifidobacterium* are capable of colonizing anaerobic hypoxic solid tumors that are poorly vascularized, invade islands of microinvasive tumor within normal brain tissues that are often not accessible to drugs and activate the immune system against cancer.

## MICROBIAL-BASED CANCER THERAPY FOR LMICS

Microbial-based cancer therapy may provide new opportunities, because the relative simplicity of microbial culturing, microbes have the potential to be self-regenerating cancer therapeutics, the ultimate sustainable cancer treatment. Humans have cultured bacteria for millennia for the fermentation of milk, bread, alcoholic beverages, for food preservation and for other applications. Such global experience in microbial culturing may offer capabilities to produce new microbial-based cancer treatment opportunities for cancer patients worldwide.

## MICROBIAL-TUMOR INTERACTION

Tumor-microbial interactions are very complex and not well understood. Challenges include potential pathogenicity and safety issues, microbial specificity and tumor targeting, microbe delivery to the tumor, immune system clearance and inactivation of therapeutic microorganisms and other challenges. Multiple mechanisms applied by tumors to evade host immune surveillance, including programmed cell death protein 1 (PD-1) and the induction of the checkpoint molecules cytotoxic T lymphocyte-associated protein 4 (CTLA-4). Such tumor cells transformation and growth require alterations in neighboring cells and their environment forming the tumor microenvironment (TME) of metastases and primary tumor. TME may considered “an immune privileged site, which provides protection against the host immune system” [5,6]. TME, which may also include the microbiota ecosystem in proximity to tumors, influences the tumor survival strategy that is associated with exploitation of metabolic pathways. The hosts’ s own microbiota can be utilized to treat cancer, for example *Bifidobacterium* which is facilitates PD-L1 and CTLA-4 blockade which unmask the tumor cell and promotes T-cell killing of tumor cell and may serve as “a key orchestrator of cancer therapy” [7].

**Figure:**
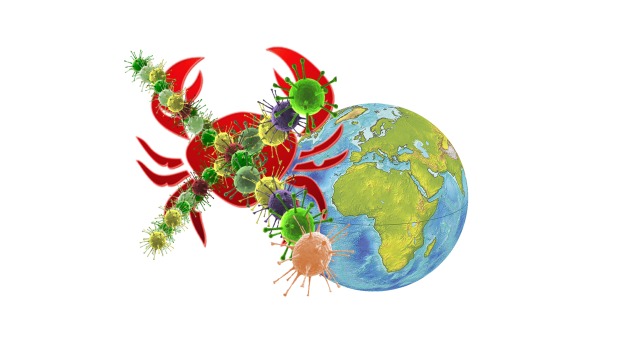
Photo: Microbial-based cancer therapy for global health: technologies to overcome global cancer health disparities (image developed by the authors, and used with permission).

Bacteria and virus gained entry and replicated only in tumor tissue because in normal tissues bacteria cleared by the immune system but not in the immune-suppressed tumor tissue.

**Figure 2:**
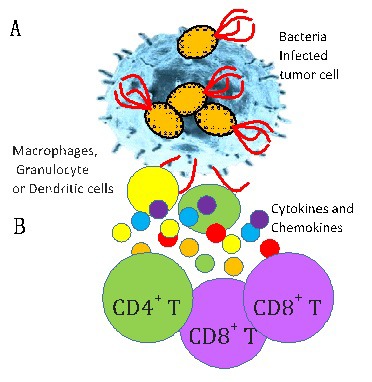
Simplified model of mechanisms for microbial-based cancer therapy. **Panel A.** Direct cell killing. Intracellular bacteria multiplication can destroy cancer cells by inducing membrane disruption, apoptosis and autophagy. Bacterial toxins can damage membrane structures or interfere with critical cellular functions. **Panel B.** Induction of the immune system. Macrophages, granulocyte or dendritic cells (DCs) accumulate in colonized tumors secrete cytokines and chemokines that activate the immune cells including recruitment of CD8^+^ T lymphocytes to eliminate primary tumors and metastases.

Microbiota microorganisms may play a major role in tumor development, cells tumorigenesis, transformation and growth require alterations in neighboring cells and their environment to support the tumor cells, forming the tumor microenvironment which may include the microbiota ecosystem in proximity to tumors. Microbiota is considered to be a key orchestrator of cancer therapy [7]. Microbiota may influence inflammation, metabolism, tissue development, immunity and control tumor response to therapy by modulating the tumor microenvironment and altering gene expression [8] and pro-inflammatory cytokines, which influences the tumor survival associated with exploitation of metabolic and immune checkpoints [9]. Bacterial antitumor localized activity (**Figure 2**) is based on the bacterial rapid intracellular multiplication, secretion of bacterial toxins which can disrupt the tumor cell membrane, cause apoptosis and autophagy. However, another effective mechanism is induction of an immune response. Bacteria induced inflammation result in tumor growth suppression and damage to cancer cells.

## APPLICATION TO CANCER THERAPY

While cancer cells evade the immune system by expressing “normal cell” checkpoints such as PD-L1 which bind to T-cell PD-1 and inhibit T-cell killing of tumor cells, commensal *Bifidobacterium* facilitates PD-L1 and CTLA-4 blockade which unmask the tumor cell and promotes T-cell killing of tumor cell and can increase effectiveness of cancer therapies. Bacteria were shown to reprogram the immune system to target cancer cells. This aspect was utilized for microbial-based cancer therapy, therapeutic bacteria stimulate the production of lymphocytes, CD8^+^ T cells, resulting in tumor clearance and eradication of established tumors. To activate the immune system, *Salmonella* expressing a “foreign” antigen (*Vibrio vulnificus* flagellin B (FlaB) was used to induced tumor suppression, bacterial colonization which is associated with TLR5-mediated host reactions and to activate the immune system against tumors harboring this *Salmonella* [10]. *Listeria monocytogenes* was engineered to deliver isotopes for targeted radiotherapy [5,11] and tumor-specific antigens enabling activating tumor-specific cytolytic T lymphocytes (CTL) inducing long-lasting anti-tumor-specific CTL responses. However, despite recent progress, many scientific gaps remain, and microbial-based cancer therapy is scientifically and clinically a very challenging field.

## APPLICATIONS TO CANCER PREVENTION

While cancer therapy is an important element in reducing cancer mortality, preventing cancer is probably the most critical and cost-effective element for cancer control worldwide. 15%-20% of cancer cases are due to microbial pathogens [12], animal studies have demonstrated that gut commensal microbiota can interfere with cancer progression and is believed that perturbed gut microbiota, known as dysbiosis, is responsible for a larger number of cases of malignancies.

Commensal microbiota can act as a tumor suppressor, but also as a promoter of oncogenesis, in this way microbiota have a widespread influence in preventing or causing malignancies [13].

Gut commensal microbiota can exert a positive effect in immune cells’ response and inflammation through effects on metabolic capacity and microbe-derived molecules such as butyrate and propionate that have shown anti-cancer activity. These metabolites can modulate the immune system and initiate an immune response preventing tumor development. For example, butyrate has shown in vivo and in vitro anti-tumor effects in colorectal cancer and lymphoma. Liposaccharide found in the membrane of gram-negative bacteria, can activate cell surface receptors triggering a T cell-mediated response against cancer development. In addition, bacterial-derived B vitamin compounds can stimulate host’s immunosurveillance.

Evidence supporting the role of the human gut microbiome in cancer prevention, Microbiota microorganisms such as *Akkermansia muciniphila*, *Bifidobacterium spp*., *Faecalibacterium* spp, and *Bacteroides fragilis* were shown to have regulatory effects on PD-1, PD-L1, and CTLA-4 blocked anticancer therapy outcome. It was demonstration that *Bifidobacteria* exhibit effects on the development of cancer through several mechanisms such as fermentation, biotransformation and strengthening the mucus barrier. In addition, *Bifidobacteria* can enhance response to cancer immunotherapeutic PD-1 blockade [14]. Therefore, altering the microbiome using an oral microbiome diet supplement may alter the gut flora enchanting our ability to prevent tumorigenesis in addition to increase effectiveness of cancer therapies discussed above.

## NCI/NIH RESEARCH INITIATIVES ON BACTERIA-BASED CANCER THERAPY

Motivated by the need to target solid tumors and in light of recent scientific progress, the National Cancer Institute (NCI) of the US National Institutes of Health (NIH) for the first time held a conference in 2017 on microbial-based cancer therapy [15] which led to the publication of a white paper on the topic [16] and to the initiation of two new NCI funding opportunities to stimulate research on bacterial-based cancer therapies for solid tumors, for conditions for which cancer therapies are inadequate and for global health. The funding opportunity PAR-19-194 is aimed to support early research without preliminary data and PAR-19-193 [17] to support more advanced research, specifically encouraging preclinical research on new microbial-based cancer therapies suitable for low resource settings, especially for cancers prevalent in LMICs and compatible with local medical/health infrastructure [17]. In addition to this new initiative, NCI/NIH is already supporting research related to microbiome and other relevant aspects of cancer prevention.

However, the NCI initiative is only the first early step in a long process to introduce new cancer therapy modalities. Developing “Bugs as Cancer Drugs” will rely on attracting researchers to the field and adequately supporting them, not only in the US but also around the world. While the pharmaceutical industries are essential to bring new cancer drugs to the bedside, these industries may have limited incentive to develop low cost cancer therapies to LMICs or to control cancer drug pricing [18], which is a barrier for treatment in LMICs. Many LMICs, especially in Asia, Latin America and Africa, are emerging economies with impressive technical capabilities in microbiology, molecular biology, oncology and related fields relevant to the development of “Bugs as Cancer Drugs”. Moreover, in many of these countries, the research enterprise is supported by governments which can direct research priorities to develop affordable cancer therapy options for their populations.

In addition to the NCI initiative, there is a need to develop an international critical mass of researchers with expertise in microbiology, immunology and oncology to develop “Bugs as Cancer Drugs” and other low-cost cancer therapeutics to provide new and sustainable cancer treatment opportunities for global health.

Bugs as Drugs are potentially self-regenerated therapeutics, by cell division bugs can generate more therapeutics bugs and the host hosts’ own microbiota can be utilized to treat cancer. These capabilities may provide new cancer treatment opportunities for cancer patients in LMIC and worldwide.
